# Utility of Automated Infrared Pupillometry in Assessing the Prolonged Course of Organophosphate Poisoning: A Case Report

**DOI:** 10.7759/cureus.58872

**Published:** 2024-04-23

**Authors:** Takuya Tomioka, Keiichiro Shimoyama, Yusuke Tanino, Masaru Hirayama, Hiroshi Homma

**Affiliations:** 1 Department of Emergency and Critical Care Medicine, Tokyo Medical University, Tokyo, JPN

**Keywords:** npi, duration of organophosphate poisoning, automated infrared pupillometry, organophosphorus poisoning, neurological pupil index, automated pupillometer evaluate

## Abstract

Central and autonomic nervous system signs of organophosphate poisoning (OP), such as altered consciousness, noticeable lacrimation, and salivation, can be influenced by medications used in intensive care settings, such as atropine and pralidoxime methyl (PAM). Because of this, there are no established methods for assessing the duration of OP while receiving antidotal treatment. In the present case, we used the Neurological Pupil Index (NPi) to evaluate the duration of OP in an 82-year-old woman who attempted suicide by ingesting up to 100 mL of fenitrothion. Until hospitalization day (HD) 20, discontinuation of atropine led to the recurrence of altered consciousness, while its reinstatement resulted in improvement; this made it difficult to assess the prolongation of OP based on signs and symptoms. Until HD 20, the NPi remained at 0/0, and subsequently, it increased. Additionally, even after discontinuing atropine, consciousness, tearing, and salivation did not worsen, indicating recovery from OP. On HD 26, serum acetylcholinesterase (AChE) levels were elevated above the measurable level for the first time, following an increase in the NPi. In this case, assessing the persistence of OP based on signs was challenging because these signs improved with atropine and PAM treatment. The improvement in NPi levels coincided with an improvement in poisoning, suggesting that NPi is useful for evaluating the duration of OP. NPi is noninvasive and sensitive compared to AChE, which is used to gauge the persistence of OP and could be used to allow earlier cessation of medication and guide appropriate treatment durations.

## Introduction

Organophosphates are widely used as insecticides and in the treatment of glaucoma and myasthenia gravis. Their ease of accessibility has led to instances of ingestion for suicidal purposes, and they have also been used in terrorist attacks. Globally, an estimated three million people are exposed to organophosphates annually, resulting in up to 300,000 deaths and necessitating the establishment of appropriate treatment strategies [1,2].

Organophosphates act as irreversible acetylcholinesterase (AChE) inhibitors, leading to cholinergic syndrome due to the excessive accumulation of acetylcholine at nerve synapses [3]. Organophosphate poisoning (OP) induces central nervous system and autonomic signs and symptoms; central nervous system signs include coma and respiratory arrest, while autonomic signs include muscarinic signs such as bradycardia, lacrimation, salivation, and diarrhea, along with nicotinic signs such as mydriasis, tachycardia, and weakness. Antidotes, such as atropine and pralidoxime methyl (PAM), have been suggested as treatments for OP [4-6].

Organophosphates have a half-life of approximately five hours; however, because of their high lipid solubility and large distribution volume, they distribute widely in tissues. Factors such as age and renal function can influence their toxicity, leading to prolonged signs [7,8]. Moreover, pupil examination, tearing, salivation, and gastrointestinal signs are influenced by antidotes and antidiarrheal agents, making it difficult to evaluate the duration of poisoning [9]. Because of this, the length of poisoning duration following drug administration remains unknown, and assessment techniques are lacking.

The automated infrared pupillometry NPi®-200 device (NeurOptics Inc., California, United States) objectively measures and quantifies the pupillary reflexes. The Neurological Pupil Index (NPi) assesses neurological severity on a numerical scale (0-5), flagging abnormalities in case of scores < 3 or with a left-right difference of ≥ 0.7 [10]. This report presents a case in which the NPi was assessed using an automated pupillometer NPi-200 to evaluate the persistence of OP.

This article was previously presented orally at The Japanese Society of Intensive Care Medicine on March 3, 2023.

## Case presentation

Patient information

An 82-year-old woman with no significant medical history or history of psychiatric or other medication treatment attempted suicide by ingesting an unspecified amount of fenitrothion (Sumithion) up to 100 ml, leading to altered consciousness. She was discovered by family members who called the emergency services and the police. An empty 100 ml bottle of Sumithion was confiscated by the police from the site; however, the exact quantity consumed could not be verified.

Clinical findings and course

**Figure 1:**
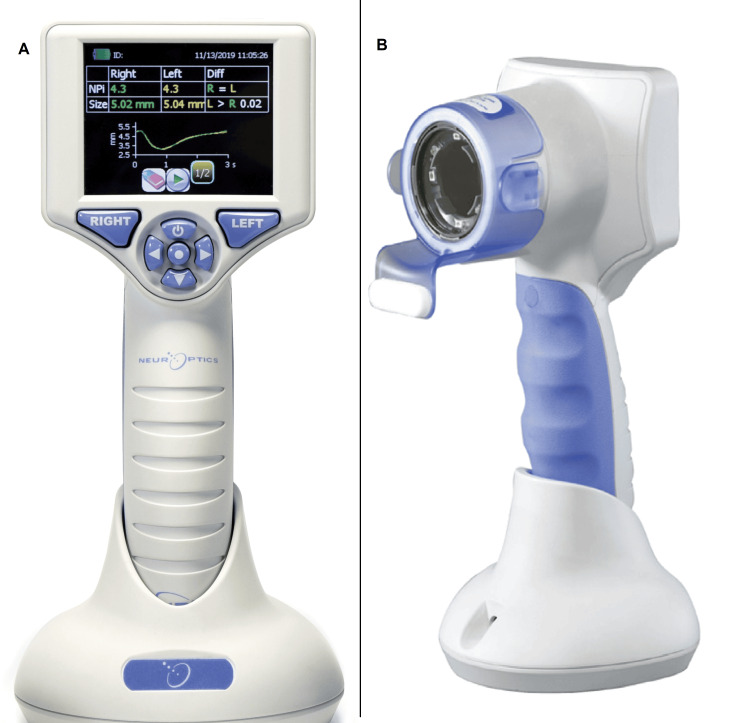
Back (A) and front (B) view of an automated infrared pupillometry instrument (NPi®-200). The screen shows representative data, not of the patient. The images were provided by IMI Co., Ltd. (Tokyo, Japan) and the permission to use the images of NPi®-200 for this report was obtained from NeurOptics Inc. via IMI Co., Ltd. No equipment loan or funding was received.

Upon arrival, the patient presented with a Glasgow Coma Scale (GCS) score of E1V2M4. Her vital signs were as follows: pupils, 3 mm/3 mm (right/left); respiratory rate, 20 breaths/minutes; saturation of percutaneous oxygen, 99% (with 10 L/min oxygen through a reservoir mask); heart rate, 110 beats/minutes; and blood pressure, 158/74 mmHg. An automated infrared pupillometry instrument (NPi-200) measured the NPi 1.5/1.5 (right/left) (Figure 1). Although the airways were patent, she exhibited shallow breathing. Physical examination revealed diarrhea and vomiting but no tearing or salivation. We noticed a petroleum odor and we removed all her clothing in the emergency room.

**Figure 2:**
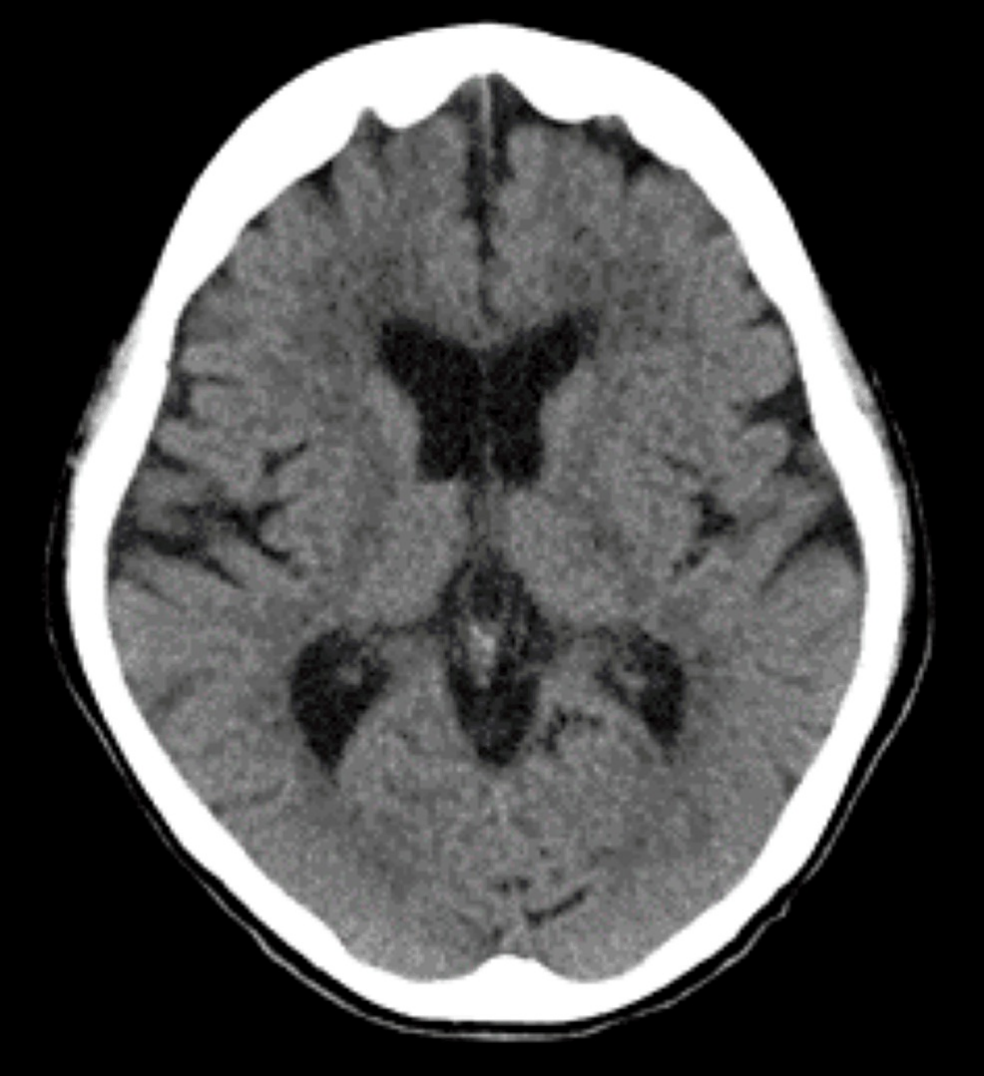
Head computed tomography on admission reveals no intracranial lesions or diseases causing the altered level of consciousness.

The patient was intubated for airway management purposes using 2.5 mg midazolam, 50 μg fentanyl, and 30 mg rocuronium by intravenous (IV) administration. The patient progressed to bradycardia with positional changes. Bradycardia improved with atropine administration; however, hypotension persisted, necessitating norepinephrine (IV) (0.1 μg/kg/minute). The initial blood test results are shown in Table 1. Red blood cell cholinesterase was not measured because our institution lacks the means to test for it. Computed tomography of the brain revealed no apparent intracranial cause of altered consciousness but indicated intestinal fluid retention (Figure 2). A drug screening test (Signify® ER; Alere San Diego, Inc., California, United States) was also performed. Amphetamine, barbiturates, benzodiazepines, cocaine derivatives, cannabis, methylenedioxymethamphetamine, opioids, oxycodone, phencyclidine, propoxyphene, and tricyclic antidepressants were not detected, and testing was not conducted for acetaminophen, salicylate, and ethanol.

**Table 1:** Blood test results on initial presentation pH: potential of hydrogen; PaO_2_: partial pressure of oxygen; PaCO_2_: partial pressure of carbon dioxide; SpO_2_: saturation of percutaneous oxygen; HCO_3_: bicarbonate

Test	Results and Units	Normal Range
pH	7.26	7.35 - 7.45
PaO_2_	214 Torr	75 - 100
PaCO_2 _	26.1 Torr	35 - 45
SpO_2 _	97.60%	92 - 98.5
HCO_3 _	11.5 mEq/L	20 - 26
Lactate	7.9 mmol/L	0.5 - 1.98
Anion gap	23.4 mEq/L	10 - 14
Creatinine	1.24 mg/dL	0.46 - 0.79
Urea nitrogen	20.9 mg/dL.	8.0 - 20.0
Acetylcholinesterase	below detectable limits	201 - 421

Based on medical history and physical findings, a diagnosis of OP from fenitrothion was established. The patient was admitted to an intensive care unit. Given the life-threatening symptoms of OP such as respiratory and circulatory depression, the patient was administered activated charcoal. Although activated charcoal poses some risk of aspiration during intubation and may have a few harmful effects on the human body while adsorbing toxins, we felt the potential benefits might outweigh the risks.

Subsequent course

Within five hours on hospital day (HD) 1, the patient's level of consciousness deteriorated to GCS E1VTM1, noticeable tearing and salivation occurred, and NPi declined to 0/0 (right/left) despite pupil measurements of 3 mm/3 mm (right/left). Since the effect of atropine was weak, after a loading dose of 1,500 mg PAM followed by continuous infusion at 400 mg/hour (10 mg/kg/hour), oral secretions decreased, and vasopressor support became unnecessary. Consciousness improved to GCS E3VTM5, although the pupils remained at 3 mm/3 mm (right/left), and NPi remained at 0/0 (right/left). Considering the significant effects of PAM, continuous administration was initiated. Additionally, there were no signs suggestive of pain, and anesthetics were not used except during intubation to rule out drug-induced consciousness disturbance.

**Figure 3:**
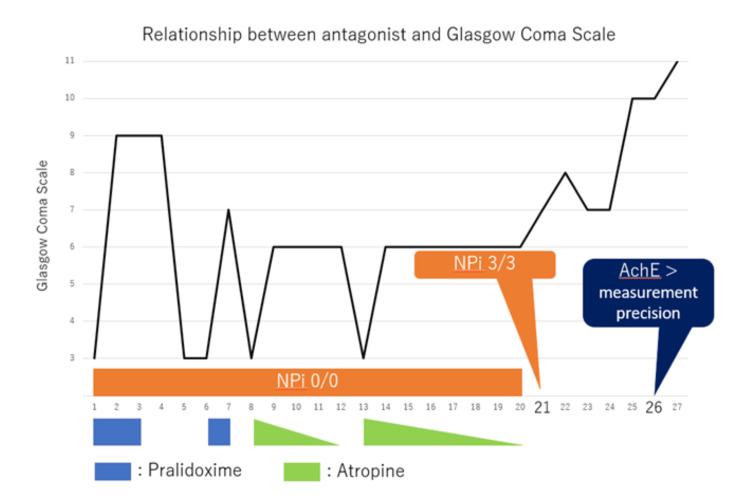
Relationship between antagonists and the Glasgow Coma Scale. Following the improvement in NPi, medication administration became unnecessary. Subsequently, there was a delayed increase in AChE. AChE: acetylcholinesterase; NPi: Neurological Pupil Index.

The above process is illustrated in Figure 3. After surpassing the half-life of fenitrothion, continuous PAM administration was stopped on HD 3, resulting in the recurrence of altered consciousness to GCS E1VTM1 on HD 5, while NPi remained at 0/0 (right/left). The significant extension beyond the half-life of the drug and differential diagnoses, including nonconvulsive status epilepticus and encephalitis, were considered; however, portable electroencephalograms showed no abnormal epileptic waves. Magnetic resonance imaging of the brain performed on HD 10 showed no specific findings.

On HD 6, consciousness remained at GCS E1VTM1, accompanied by increased oral secretions. Suspecting prolonged OP due to the recurrence of physical findings, a PAM dose of 400 mg/hour (10 mg/kg/hour) was initiated, resulting in an improvement in consciousness to GCS E2VTM4 and a decrease in oral secretions.

PAM was continued over the next 24 hours until HD 7; physical findings such as altered consciousness and oral secretions improved, although pupil measurements remained at 3 mm/3 mm (right/left), NPi consistently remained at 0/0 (right/left), and AChE levels remained below detectable limits. Discontinuation of PAM administration on HD 7 resulted in worsened diarrhea by HD 8, along with marked tearing and salivation, with consciousness returning to GCS E1VTM1. Considering aging [11], atropine at 0.0625 mg/kg/hour was started, leading to an improvement in consciousness to GCS E1VTM5 and a reduction in tearing and salivation. As the patient continued to experience altered consciousness and excessive airway secretions due to persistent OP, a tracheostomy was performed on HD 9. Subsequently, atropine was tapered and discontinued by HD 12; however, on the same day, consciousness deteriorated to GCS E1VTM1, accompanied by the recurrence of significant tearing and salivation. Atropine re-administration improved consciousness to GCS E1VTM5, suggesting persistent OP. Subsequently, the atropine dose was tapered, and by HD 20, its administration was discontinued.

Until HD 20, atropine discontinuation led to the recurrence of altered consciousness, and its reinstatement resulted in improvement, making it difficult to assess the prolongation of OP based on signs. Until HD 20, pupil size remained unchanged at 3 mm/3 mm, and the NPi consistently remained at 0/0 (right/left).

From HD 21 onward, the NPi increased to 3/3 (right/left), and even after discontinuing atropine, tearing and salivation did not worsen. Consciousness remained stable at GCS E2VTM6, indicating recovery from OP, although AChE levels remained below the detectable limit. On HD 26, the AChE levels rose to 13 U/L following an increase in NPi. Subsequently, both NPi and AChE levels continued to increase. On HD 43, the tracheostomy was closed, and consciousness improved to GCS E4V5M6. Rehabilitation for post-intensive care syndrome was continued in the general ward, and the patient was transferred to another hospital on HD 81.

## Discussion

In this case, the signs of OP (altered consciousness, diarrhea, tearing, and salivation) improved with atropine and PAM administration, making it challenging to evaluate the prolongation of poisoning based on signs alone. NPi consistently remained at 0/0 (right/left) throughout poisoning, and the improvement in NPi coincided with an improvement in OP. While other clinical observations lacked precision, NPi allowed for the evaluation of the persistence of OP.

NPi is derived from seven indicators: initial pupil size, post-light reflex pupil size, constriction ratio, mean constriction velocity, maximum constriction velocity, latency to the onset of constriction in response to light stimulation, and mean dilation velocity. Although the subjective assessment of central nervous system-related findings through pupil examination and light reflex is customary, the ability to quantify these objectively makes NPi particularly useful in neurological intensive care for monitoring intracranial pressure, prognostic evaluations in post-cardiac arrest syndrome, determining cutoffs, and longitudinal observations [12,13].

The evaluation of pupillary responses by automated infrared pupillometry with the NPi-200 involves the dual antagonistic control of the autonomic nervous system, made up of the sympathetic and parasympathetic nervous systems. Due to the effects of organophosphates, the concentration of the neurotransmitter acetylcholine increases, and in cases where the parasympathetic nervous system becomes dominant, the maximum constriction and dilation velocities of the pupils during the light reflex increase [14]. The maximum constriction velocity is solely controlled by the parasympathetic nervous system; when it dominates, the maximum constriction velocity is prolonged [15]. Consequently, NPi (which is influenced by the maximum constriction velocity) is affected.

Organophosphates affect the central and autonomic nervous systems. Atropine acts only on the autonomic nervous system as a competitive antagonist of the muscarinic acetylcholine receptors. PAM also acts as an AChE reactivator. A large dose of pralidoxime is required to antagonize the central nervous system effects of organophosphates, implying the limited susceptibility of the central nervous system to the effects of antagonists [16]. In the present case, the autonomic signs of OP improved with atropine and PAM; however, the central nervous system signs evaluated using NPi did not improve. This suggests that the central nervous system is less susceptible to the effects of antagonists.

Assessment of the duration of OP generally relies on AChE levels in addition to pupil examination. AChE exists in two forms: red blood cell AChE and serum AChE (also known as butyrylcholinesterase). The patterns of inhibition and recovery of the activities of these two AChE are highly variable according to the organophosphate and in different individuals [17]. Also, in routine clinical practice, serum AChE is typically measured; however, because AChE activity depends on the inhibition and regeneration rate caused by organophosphates, it has been suggested that signs of poisoning may not correlate with AChE levels [18,19]. In this way, it is difficult to claim that AChE is reliable in the assessment of OP, and thus, a more sensitive indicator is required. In this case, there was no observed decline in the level of consciousness after the discontinuation of the antagonists after the 21st day of treatment. While the NPi showed recovery on HD 21, the recovery of AChE was delayed until HD 26, suggesting that NPi is a more sensitive pathological marker than AChE.

The pupil findings improved with antagonists and were subject to interrater variability; in addition, AChE assessment requires daily blood sampling, and AChE levels may not correlate with poisoning signs. These problems make it challenging to accurately evaluate the duration of OP. NPi is noninvasive and allows for frequent measurements, suggesting its potential sensitivity in detecting the persistence of OP. Early recognition of detoxification through NPi assessment could lead to the timely cessation of antidote administration, contributing to the establishment of appropriate treatment periods.

Potential limitations and implementation

In recent years, the administration of atropine for OP has suggested the need for individualized dosing based on signs rather than based on traditional weight-based methods [4]. Similarly, regarding PAM, reports have indicated the ineffectiveness of low-dose administration and the efficacy of high-dose administration [1,20], which might have influenced the NPi improvement in this case. This indicates that inadequate dosing in this case might have affected the improvement in NPi.

Additionally, the price of this device is 970,000 yen (approximately $6,400). Generally, the NPi-200 is used for the assessment of the pupil in critically ill patients, but its utilization varies by region and facility. It is widely used in some large medical facilities and intensive care units, and adoption depends on the hospital's equipment and financial situation. In Japan, it is used in 152 facilities, but its adoption is still considered low globally.

As this is a case report of a single patient, large-scale investigations are required to determine the generalizability of NPi efficacy in evaluating OP.

## Conclusions

The pupil findings improve with antagonists and are subject to interrater variability. Moreover, AChE assessment requires daily blood sampling, and AChE levels may not correlate with poisoning signs. These problems make it challenging to accurately evaluate the duration of OP. The current case indicates that NPi appears to be useful for evaluating the duration of OP. NPi can gauge the persistence of OP noninvasively and sensitively, potentially leading to early cessation of medication or appropriate treatment duration settings. This report pertains only to a specific patient and further large-scale studies are needed to determine the generalizability of the effectiveness of NPi in evaluating OP.
